# Older Adults’ Perceptions of ICT: Main Findings from the Technology In Later Life (TILL) Study

**DOI:** 10.3390/healthcare7030086

**Published:** 2019-07-04

**Authors:** Hannah Ramsden Marston, Rebecca Genoe, Shannon Freeman, Cory Kulczycki, Charles Musselwhite

**Affiliations:** 1 Health and Wellbeing Priority Research Area, School of Health, Wellbeing and Social Care, The Open University, Milton Keynes, Buckinghamshire Milton Keynes MK7 6BJ, UK; 2 Faculty of Kinesiology and Health Studies, University of Regina, Regina, SK S4S 0A2, Canada; 3 Faculty of Nursing, University of Northern British Columbia, Prince George, BC V2N 4Z9, Canada; 4 Centre for Innovative Ageing, Swansea University, Swansea SA2 8PP, UK

**Keywords:** technology, rural ageing, qualitative research methods, gerontechnology, privacy, intergenerational, social connectedness, community networks

## Abstract

Technology is entwined in 21st Century society, and within the lives of people across all ages. The Technology In Later Life (TILL) study is the first piece of work contributing to the impact, behavior, and perception of technology use, by adults aged ≥70 years, residing in rural and suburban areas. TILL is an international, multi-centred, multi-methods study investigating and conceptualizing how various technologies impact the lives of older adults; residing in urban and rural locations in the United Kingdom (UK) and Canada. This in-depth study recruited 37 participants via a multi-methods approach. Analysis of the findings ascertained two overarching themes: facilitators of technology use (i.e., sharing of information and feeling secure), and detractors of technology (i.e., feelings of apprehension of use). Proposed recommendations include promotion of technology from a strengths-based perspective focusing on positive opportunities technology to improve health and wellbeing, creating a peer support network to assist with learning of new technology, and the need to examine further how intergenerational relationships may be enhanced through the use of technology. The distinction of these themes narrates to the originality of this initial study and milieu of recruited participants, intersecting across the fields of gerontology, geography, social sciences, and gerontechnology.

## 1. Introduction

The digital divide is commonly discussed when examining ownership of or access to information communication technologies (ICT), in addition to the possession of the skills and expertise required to use ICT to access information by older adults. Accessibility of ICTs is often dependent upon ICT literacy. For example, having limited knowledge to execute an Internet search may also reduce users’ access [1].

The debates surrounding the digital divide have been ongoing for nearly thirty years, in a bid to enhance the quality, access, and equality of ICTs and information, while empowering users from all socio-economic areas [2]. With the former, greater social interaction and engagement of civic involvement occurs, which in turn reduces social connectedness, increases knowledge and skills, and facilitates communities and individuals to better their themselves and their families [3]. Yet, in 2003, the authors of [4] noted that with the rise of ICT and technology developments, there is the possibility that such innovations could increase inequality, rather than improve and restructure exiting concerns. As the Internet formed part of the earlier debates of the digital divide aiming to understand the diffusion of the internet, access to new technologies was accessible to individuals from higher social denominations and who were educated [5].

Previously, several models and frameworks have proposed a myriad of definitions, and conceptualizations of what constitutes the digital divide. DiMaggio and Hargittai [6] describe the five facets of digital inequality: (1) equipment, (2) autonomy of use, (3) skill, (4) social support, and (5) the purpose for which the technology was used. Meanwhile, Selwyn [7] propounded the digital divide through four stages: (1) formal/theoretical access to ICTs and content, (2) effective access to and use of ICTs and content, (3) engagement with ICTs and content, and (4) outcomes or consequences. Moreover, van Dijk [8] suggested a model comprising of four key facets associated to access: (1) motivational access, (2) material access, (3) skills access, and (4) usage access. Yet, a digital divide index was defined and proposed by the authors of [9], comprising of five elements: (1) infrastructure access, (2) affordability, (3) use, (4) social and governmental constraints/support, and (5) sociodemographic factors. These varying models, definitions, and proposals not only offer, but also broaden perspectives to research conducted over a period of three decades.

However, as early as 2000, scholars [10] questioned whether there was a digital divide at all, and how this digital divide was made up—through income, education, access, skills, and/or based on geographic location (rural vs. metropolitan). For example, Brady notes the following:

“Computers and Web appliances are now relatively cheap, and free Internet access is available in many areas. Even lower income families could find a way to get wired if they viewed it as a high enough priority.” (Brady, 2000)

Rooksby and colleagues [10] note this perspective is not significant and fails to recognize differences between and across those who have and those who do not, or those individuals who are rich and poor. Similarly, Compaine [11] supports this notion between the haves and have-nots, while others [12,13] perceive the notion of the digital divide is ‘bridging itself’ [10], and thus it is the responsibility of governments to offer financial assistance to support access to government information online. For example, Rooksby et al. [10] propose that governments should match funds in conjunction with the private sector to align ICTs, and regional and distribution centres should be developed to facilitate access and to monitor the gaps in Internet access. Over the last two decades, governments across the Western world have been attempting to reduce the digital divide through different initiatives and collaborations. Such initiatives in the UK aimed to tackle issues ranging from accessibility of ICT to infrastructure, roll out, and improvement of broadband. Yet, as we have seen through previous scholarly work, areas of interest have also included income, age, gender, and location; while these factors are still areas of interest, the development of technology since the turn of the twenty-first century has been phenomenal. This is particularly so, when governments are changing their behavior to offering information access and services.

While the digital divide comprises several factors including access, ability, and affordability, for others, additional factors may also play a role in the digital divide including, gender [14], age [15,16], income [17], education [18,19] ethnicity, and geographic location [20].

### Literature Review

Across the UK, Internet upgrades have taken place with the notion to “reduce inequalities in Internet access, defined geographically: that is, disparities in access between different regions” [21]. Previously, infrastructure and access have been the primary foci of the digital divide [11,22] and while the provision of equipment is important, having the skills, knowledge, and digital ICT literacy to use ICT must also be considered. The use and deployment of ICT may have a profound effect on their lives of many people across social classes, countries, and socioeconomics. Yet, without the knowledge of how to use ICT, bridging the gap will remain limited, resulting in information inaccessibility, disengagement, and disinterest [23,24].

A growing area of work is the use of the Internet by older adults [1,25,26,27,28,29,30,31]. In 2016, more than two-thirds of Americans aged 65 and older used the Internet, and 51 percent had high-speed Internet at home [32]. Zickuhr [30] noted that 69 percent of adults aged over 65 years owned a cell phone, 48 percent owned a desktop computer, 32 percent owned a laptop, 11 percent owned an e-reader, and eight percent owned a tablet [31]. However, within the nontechnological users of the study, 68 percent recognized that their self-confidence was a problem. Furthermore, those individuals also acknowledged that they would benefit from learning how to use ICTs in their daily lives [30]. Anderson and Perrin [32] reported 40 percent of American older adults owned a smartphone in 2016, while smartphone usage was declining in adults aged ≥75 and older.

Currently, research has focused on the existing and prospective role(s) ICTs may have in later-life as a means of reducing social isolation and loneliness [25,28,33,34,35,36]. Additionally, and since 2010, this work has aimed to understand the needs and requirements of older adults, identifying that communication across generations is important, while also acknowledging, for some, that having the skills and knowledge to understand how to access ICTs (i.e., Skype) is also an area that needs further exploration.

While contemporary research has explored the digital divide from the standpoint of leisure and engagement by users and non-users of ICT [37], the reduced engagement and interaction by users and non-users may vary based on one’s personal choice(s) and interests [38]. However, Ihm and Hsieh [39] note how access to ICTs is greatly reduced in later life compared with in younger users. Thus, this is problematic for older adults who did not have the opportunity to use ICTs in their previous employment and/or careers; thus greatly increasing their limitation to access, knowledge, services, and financial constraints (i.e., online banking) [40].

In a recent study conducted in the Czech Republic, Klimov and colleagues [41] explored whether age impacted on Internet use among 432 older people who were both active and passive users of technology. Respondents’ age ranged between 55 and 94 years. Gender was not equal, comprising of 73 percent female and 17 percent male. A total of 15 participants were aged 85+ years, the majority of participants were aged between 65 and 74 years (*n* = 257), comprising of 188 females. The findings identified that participants between 55 and 74 years do use the Internet, while adults aged 74+ years spend less time using the Internet [41]. Similar findings were identified in earlier studies [31,42,43]. Additionally, respondents reported using the Internet for communication purposes, specifically using email, followed by online banking, Skype communication, and sharing photographs. Similarly, Choi and DiNitto [44] reported that their respective participants engaged in similar Internet-related activities (email/SMS communication, online shopping/banking and paying bills, health related tasks). However, Klimova et al. [41] noted their study was conducted in one specific area of the country and did not explore or take into consideration participants’ education levels or socio-economic backgrounds. Conversely, Neves et al. [28] undertook a participatory design approach to developing an “accessible communication app” (page 1) primarily aimed at frail and institutionalized older adults, as a means of reducing social isolation and loneliness. The results from this study identified “that technology adoption is a based on a complex set of interrelated factors: social, attitudinal, physical, digital literacy, and usability” (page 1). Further considerations were noted by Neves and colleagues [28], suggesting there are differences and expectations of communication (i.e., style, feedback, availability) across generations.

Ivan and Hebblethwaite [45] aimed to understand social media use (i.e., Facebook) by grandmothers in Romania and Canada, in a bid to ascertain how intergenerational relationships of grandparent and grandchild are built by sharing photos with one another. Conversely, previous scholarly activity in this domain has explored the relationship between gender and ICT, exploring how and why individuals use ICTs in both younger and older adults [46,47,48,49]. The findings by Lian and Yen [48] show how performance and the social influence are strong factors to using ICT by older adults, which in turn are similar to the factors and social influences of younger adults. Yet, the respective study concluded there was no gender difference relating to the barrier and enablers of online shopping.

While we have witnessed a growth of scholarly research focusing on health and rehabilitation within the field of game studies [50,51,52,53], what has been lacking from contemporary research is understanding the technology experiences of respective participants recruited to respective studies. Marston, Kroll, Fink, de Rosario, and Gschwind [54] reported technology use by participants who primarily used technology for communicating via email, searching for information, text processing, and online shopping. While some of the participants did report using social media platforms such as Facebook and Google+, Marston and colleagues [54] suggested greater work is needed in the form of in-depth qualitative interviews and focus groups to ascertain the needs and issues surrounding current ageing populations and the impact technology has on their daily lives.

While there is a growing body of work focusing on the impacts of ICTs in later life, there is a need to explore and understand the barriers and enablers of ICTs by adults aged 70> years residing in rural and urban geographical locations to ascertain and understand the needs and requirements of existing age cohorts.

The significance of the Technology In Later Life (TILL) study intersects across the fields of gerontology, social sciences, geography, and gerontechnology, resulting in the contribution of new knowledge and proposed recommendations to advance future work across these multi- and cross-disciplinary fields.

## 2. Materials and Methods

### 2.1. Aims and Objectives

The Technology In Later (TILL) project aimed to examine the experiences of older adults aged 65+ years with technology, exploring how they embraced (or did not embrace) various types of ICT, and what the barriers and/or challenges faced by this older generation are in the take-up and continued use of ICTs in later life. Subsequently, the team sought to identify implications of using ICTs for current and future aging populations in rural and urban geographic locations. A multi-methods approach was adopted to enable the project to undertake “the inquiry on the assumption that collecting diverse types of data (quantitative data via one of two online surveys and focus group data) best provides an understanding of a research problem” [55] (pg. 21).

### 2.2. Participants

Thirty-seven participants were recruited via education facilities, public places (e.g., a library notice board, mailing lists), or seniors’ centres such as Age UK Milton Keynes. Participants were included in the study if they used technology (for any amount of time), were 65+ years old, and lived in and around the surrounding respective study sites. There were 20 rural participants (McBride, British Columbia, (BC), Canada *n* = 10 and Wales, UK *n* = 10) and 17 urban participants (Regina, Saskatchewan (SK) Canada *n* = 6 and Milton Keynes, UK *n* = 11). Table 1a displays the overall demographics of participants and across each the UK and Canada, while Table 1b displays the demographics of participants from the perspective of rural and urban locations. The age range of participants was between 67 and 89 years, with a mean age of 77.

### 2.3. Recruitment

Participant recruitment procedures were tailored as required on a site by site basis. Participants in the Milton Keynes area were recruited from the Age UK Milton Keynes Centre, while for the two sites in South Wales, participants were primarily recruited from the Older People’s Forum comprising of 1000+ older people living across Wales. In Canada, participants from the city of Regina were recruited through flyers and posters across the local area, while participants in the town of McBride were recruited through an advertisement posted at a local senior’s advocacy centre and in the community monthly newsletter, as well as through a radio interview for a senior’s program on a local radio station.

A flyer and poster were created detailing the project study and aims—each tailored to include the contact details for each study site/partner. A mailing list script was created and submitted as part of the recruitment documentation. The mailing list script was primarily utilized by Swansea University. Participants were included in the study if they were users of technology and were ≥70 years old.

### 2.4. Study Locations

The Technology In Later Life (TILL) project is an international, multi-centred exploratory study comprising of two countries (UK and Canada) and four sites (two rural and two urban). Sites were selected based on the differences in physical environment, size of population, and accessibility to different ICTs. Additional rationale for choosing the selected sites is based on the location of the researchers of this initial study. Milton Keynes, located in the county of Buckinghamshire, was the urban site in the UK, and in 2013, was reported to have a population of 255,700 [56]. The urban site selected in Canada was Regina, the provincial capital of Saskatchewan, which has a population of 230,725 people [57]. The main industries in Regina are mineral resources and agriculture.

The rural locations in the UK consisted of the two small towns of Cwmtwrch and Ystalyfera, located less than 3 miles apart from each other in South Wales some 15 miles north of Swansea. Previously, Ystalyfera was industry-based with a large tin works that was closed 1946. Ystalyfera is located three miles east of Cwmtwrch and the population currently stands at just under 5000 people [58]. Cwmtwrch is a former industrial area, which used to have coal mines and iron works that are now all closed, and the current population is 2074 [59]. Presently, it is now largely agrarian in nature and is on the edge of the Brecon Beacons, one of the UK’s national parks. In Canada, the town of McBride, British Columbia (BC) was the selected rural site. McBride, located in the Robson Valley region near the Rocky Mountains, is a small farming and forestry town and has a population of 660 people [60].

### 2.5. Ethical Approval

Ethics approval was granted by all four institutions by mid-September 2015 and participant recruitment and data collection were conducted between autumn 2015 and February 2016 (Open University: HREC/2015/2028/Marston/1; UNBC: E2015.0714.061.00; Swansea University: Granted-no code given; University of Regina: REB#2015-113). For each phase of data collection, informed consent was obtained. Prior to completing the online survey, participants were required to tick/check the consent box, which enabled them to complete the survey. All participants who completed the online survey also agreed to participate in a focus group discussion. Verbal and written informed consent were completed by each participant prior to the focus group commencing. Prior to the start of the focus groups, each participant signed an informed consent form.

### 2.6. Procedure

#### 2.6.1. Online Survey

The online survey deployed in the TILL project was created using Google (Docs) survey^©^, and shared via a link to all participants who had agreed to take part in the study. The online survey was voluntary to complete by each participant across the four sites and was required to be completed prior to attending a scheduled focus group. Participants were required to confirm their consent at the beginning of the online survey by checking/ticking the box.

The survey was a later iteration of an earlier survey [54,60]. The 80-item survey covered eight domains: (1) Technology use; (2) Internet ownership and use; (3) Social networking; (4) Digital device ownership; (5) Purchasing patterns; (6) Quantified Self and lifelogging; (7) Information sharing and privacy issues; and (8) Demographics. A copy of the survey is available in the supplementary data.

Some survey items were amended to reflect minor differences in the British and Canadian English language. For example, in the UK, participants would be asked to “tick the box”, whereas a Canadian participant would be asked to “check the box”; or a Canadian participant would use the term “gas”, whereas a UK participant would say “petrol”.

#### 2.6.2. Focus Groups

Each site aimed to recruit 10 participants and potentially split into two groups of five or six people to ensure the fluidity of discussion, ease of transcribing, and that no one was over shadowed when describing their ICT experiences.

A total of six focus groups were conducted in both the UK and Canada. Sixteen participants were recruited in Canada. In the UK, a total of 21 participants were recruited. Two focus groups were conducted in an urban location (Milton Keynes, Buckinghamshire, UK) and two focus groups were conducted in rural locations (South Wales, UK), comprising of 20 participants residing in rural locations and 17 in the urban location. In Canada, two focus groups were conducted in a rural sites (McBride, BC, Canada), and one focus group was conducted in an urban site (Regina, SK, Canada).

A semi-structured approach was taken, and the questions focused on ICT use/ownership; rationale for using ICTs; social media habits and perceptions; privacy issues and concerns; sharing of information (e.g., why, how, type of content shared), from a traditional (pen/paper) and digital (mobile app/Facebook) standpoint; and what the future holds for ICT in society, health, and ageing populations.

Prior to the focus group discussions, all participants were shown vignettes, which comprised of negative and positive perspectives to using different technologies. The vignettes were developed by the Open University partner for a previously unrelated project and encompassed sketches/content relating to ICT use/experience in different contexts and environments. A full description of the vignettes shown to the participants prior to the focus group discussions commencing can be found at the following website (http://bit.ly/2XH5CFu). The vignettes were shown to the participants comprising of positive and negative perspectives of using different types of technology in different scenarios, yet familiar environments such as a health practitioner surgery, a home, and an office/social environment. The rationale for showing vignettes was to assist the participants in creating a basis for the discussion during the focus group sessions.

### 2.7. Data Collection and Analysis

Focus group discussions were digitally audio recorded for a minimum of 60 minutes and transcribed by an external company in the UK. A qualitative descriptive approach [61] was conducted, comprising of an investigator triangulation approach to analyzing the focus group data. Meanwhile, data analysis occurred via initial and focused coding and constant comparison, drawing from grounded theory guidelines by Charmaz [62]. For more information relating to data analysis, see the work of [26].

This approach resulted in a rich description of participant experiences. Ongoing peer checking was undertaken by research investigators to ensure the authenticity, credibility, and trustworthiness of the analysis [63,64,65]. An inductive approach was selected to generate new knowledge from the data [66] and a descriptive cross-sectional study approach was selected, as it is appropriate study design to more broadly describe participant demographics and experience from a quantitative perspective. Qualitative data were classified into categories as a way of describing the role and impact that technology plays in the lives of older adults [67]. Quantitative analysis used SPSS statistics version 24.

## 3. Results

### 3.1. Quantitative Data

Findings from the completed online surveys (*n* = 37) and focus groups will be presented in the following sections, providing insight into technology use by total population, country, and site.

Table 2 displays results focusing on participants use, access, and ownership of computers in their daily lives. The majority of participants owned a personal computer (PC) and the frequencies of participants across each country are nearly equal, but people are slightly more likely to own a PC in rural areas in the UK and in urban areas in Canada. Five participants did state they owned an Apple/Mac computer. The majority of participants accessed their computer in their own home, followed by seven participants located rurally in the UK reported to have accessed their friend’s computer and one person living in the urban location. Similarly, six participants in the UK reported to have used a computer owned by an adult child, and a further eight participants reported to have accessed a computer in a public building. However, this was not the case by Canadian participants, who primarily reported exclusively to use their computer in their own home. Most participants have been using computers for over 20 years. This is particularly so for Canadian participants living in rural Canada. Frequency of using a computer showed that the majority of participants used their computer more than once a day, with relatively equal numbers across the UK, Canada, and all locations.

There were several reasons why participants used a computer (Table 3), with the majority reporting email (*n* = 29), followed by checking facts on the Internet (*n* = 25), word processing (*n* = 24), and online shopping (*n* = 22). There were additional reasons why they used a computer ranging from online banking (*n* = 18) to using social media (*n* = 13), playing games (*n* = 10), and spreadsheets (*n* = 8), while 15 participants reported using a computer for ‘other’ reasons. However, participants did not elaborate on these other reasons. Checking facts on the Internet was more popular in Canada (13/16) than in the UK (12/21), and was especially low in rural UK (3/10). Online shopping was especially low in urban Canada (2/6) and highest in urban UK (8/11). Database and spreadsheet use was highest in urban UK (5/11) and non-existent in rural UK (0/10). Social networking on computers was higher in Canada (8/16) than in the UK (5/21) and this was further explored in Table 4, which shows that despite this, those that do use it in the UK are more likely to introduce others to it than those using it in Canada. Most people access it daily and have been using it for between 5 and 10 years’ time. The most popular reason for using social media was to stay connected to friends and children/grandchildren (Table 5). Staying connected with children or grandchildren was slightly higher in rural locations than it was in urban locations in both the UK and Canada.

Participants across the UK and Canada reported owning a myriad of digital devices, with the majority owning a mobile phone, and in particular participants residing in the urban—UK location, with a very low number in the urban Canadian area (see Table 6). Yet, from a Canadian standpoint, the majority of participants residing in the rural location reported to own a mobile phone. Equal numbers of UK and Canadian participants reported to own an Apple iPad, coupled with participants residing in respective urban locations. Six Canadian participants equally split between rural and urban locations reported to own an Apple iPhone, while one participant residing in the rural location reported to own an Apple iPad. Owning a tablet device was more popular among UK participants residing in the urban location rather than rural and Canadian participants. Owning a Fitbit/pedometer was primarily reported by UK participants living in the urban location (*n* = 2), with a further one participant in the rural location noting their ownership. Overall in the UK urban areas, more devices were owned (25, showing an average of over 2 devices per person; compared with 18 from the 10 rural UK participants; 15 from the 10 rural Canadian participants; and 14 from the 11 urban Canadian participants).

Participants were asked if they shared their information (see Table 7). The majority of participants reported to share their information because of having common interests (*n* = 16), while 11 participants reported to share their information ‘because it is fun’. This rationale was primarily conducted by UK participants living rurally (*n* = 8), compared with none living in the urban area of the UK and only two in rural Canada and one in urban Canada. Sharing information to make sure the recipient is thinking of me was exclusively a UK and almost exclusively an urban UK reason. To increase friendship was noted by seven individuals, but notably not at all in rural Canada. To start or continue a conversation was largely a UK factor and in rural areas, with only one Canadian mentioning it.

Table 8 notes that friends and family in the UK (*n* = 9) are much more likely to self-log than those in Canada (*n* = 3), especially in urban areas. In addition, self-logging on smart phones is much more prevalent in the UK (*n* = 5) than in Canada (*n* = 1). Similarly, self-logging on the computer is also more prevalent in the UK (*n* = 5) than it is in Canada (*n* = 0). Those in the UK (*n* = 7) are also more likely to consider taking up self-logging than those in Canada (*n* = 1).

**Figure 1:**
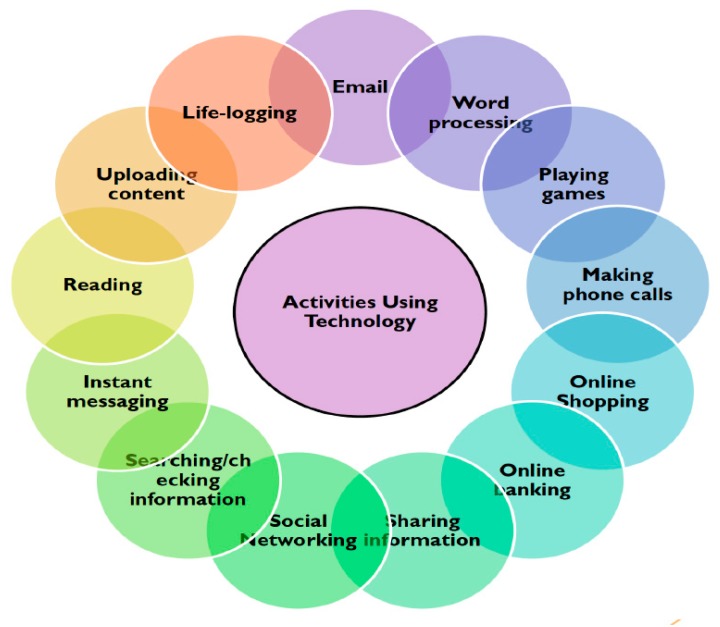
Participant reasons for using technology.

Figure 1 illustrates the myriad of activities conducted and reported by our participants during the focus groups, detailing that our participants do have a wide variety of activities that integrate ICTs and technology into their daily lives.

Data analysis of the qualitative data from the focus groups ascertained two primary themes: (1) facilitators of ICT use and (2) detractors to ICT use. Each primary theme had several associated subthemes (see Table 9).

The first primary theme, facilitators of technology use, identifies the factors that contributed to the adoption and use of technology. The second primary theme, detractors of technology use, highlights factors that impeded or limited their use of technology. We describe each theme and its corresponding subthemes in detail below (Note: participants are denoted by location and participant number; MK refers to participants from Milton Keynes, R refers to participants from Regina, W refers to participants from Wales, and McB refers to participants from McBride).

### 3.2. Facilitators of Technology Use

Participants identified several facilitators that led them to adopt or continue to use technology in their daily lives, thus demonstrating that technology use can play a positive role for participants across all study sites. Facilitators included technology learning opportunities, having access to technology, learning and sharing information, and feeling secure.

#### 3.2.1. Technology Learning Opportunities

For many participants, having the opportunity to learn how to use technology, whether in the workforce as an employee, or post-retirement in a structured learning environment, facilitated technology use. Having opportunities to learn within the workplace allowed for building skills in an environment where support was available if needed:

*My first encounter with a computer is obviously in work, latterly in work, and always had the backup support within work as well. If anything went wrong there was always somebody that you could ring and get things out, so that’s okay.*

*[MK4, female]*

Some participants described positive experiences with classroom learning geared towards seniors that provided opportunities to learn how to use different devices:

*I took […] classes …which I found to be very helpful. I think I’ve had the three iPad classes, so two iPhone classes and several computer classes, they offered years ago, and they were always very, very helpful.*

*[R3, Female]*

Taking initiative to seek out learning opportunities helped to facilitate its use. A participant from Milton Keynes noted that she had not had the opportunity to learn to use a computer in the workplace, and thus took a certificate program to learn the basics:

*…when computing was brought out and I was teaching, we never did that when we were kids or at school or training. I had to just bite the bullet and just get to it, otherwise I was going to be left behind, very much so. I did all the different things for this certificate, PowerPoint, database, spreadsheets, all the bits like that. You can figure it out. It’s not too difficult if you really set to.*

*[MK2, Female]*

#### 3.2.2. Having Access to Technology

For many participants, simply having access to technology, particularly the Internet, served as a motive for its use. For example, when technology was portable, it was perceived as being more accessible than instances when individuals were tied to a desktop. A participant from the Canadian rural site, McBride, found technology to be much more useable once she had access to high speed Internet, something that was introduced in rural areas of Canada much later than in urban areas. She stated the following:

*I just basically use [my laptop] as a nice portable machine that I can take with me and have lots of information and access to the internet. Because for the longest time I was on dialup and doing these daily emails until about a year and a half ago I could finally get a connection through the new cell tower they put in…so I am on high speed now. It’s not really high, high, high speed like you would get in the city but it is like 500 times as fast as dialup was.*

*[McB2, Female]*

With the combination of high-speed Internet access as well as a laptop, this participant found accessing the internet much easier in order to send emails and access information. Indeed, for some of these rural participants, high speed Internet was preferred such that they were willing to pay a higher cost to obtain it: *“And now we have bought an air card at great cost per month so that you are not sitting there for 10 minutes or whatever to get that dialup.” [McB3, female]*

Although for some participants, laptops were the preferred means of accessing technology, for others, a desktop, with internet access, sufficed:

*Yes. I’ve got a PC that I really like. It’s like my right arm, wouldn’t be without it. On it every day, twice a day, maybe three times a day, just to find out what emails have come in.*

*[MK1, Female]*

#### 3.2.3. Learning and Sharing Information

In addition to having access to technology in general and Internet use in particular, participants were motivated by a desire to find and share information. In particular, participants were motivated to use technology to find information related to health. A participant from Wales stated the following:

*I do use the internet to search on health subjects. You can go on, as you say, I use that too, the Mayo Clinic and I use the sites, the National Institute of Health in the US. Well that’s because that’s what I am familiar with, you know, when I lived there. But I wouldn’t go on to some of these forums that you were talking about. They are not very reliable, and they are just people expressing their views. You want evidence to support what is being said.*

*[W3, Female]*

While participants used the Internet to find information, they recognized the need to be careful with regards to the types of information they were accessing and ensuring that the information was reliable. Wearable technology was also utilized as a means of gaining information about health as participants tracked their own health behaviours, such as levels of physical activity or medication use. Participants were interested in monitoring their physical activity levels through technology:

*“Well, I personally wouldn’t mind one of these, where can you get the Fitbit?...I would like to know how many steps I am doing a day so therefore what can I do to improve?” *

*[W2, Female]*

In addition to gaining health information, participants wanted to use technology in order to communicate with others and share information. Participants across the focus groups detailed how they chose to communicate and engage with technology to keep up to date with friends and family. Using videoconferencing software such as Skype or having access to a computer in a public space enabled communication with children or grandchildren who were geographically dispersed. A participant in Milton Keynes spoke about keeping in touch with his daughter in another country by utilizing FaceTime:

*I’ve used Skype because my daughter lives in South Africa, but it’s an atrocious service because South African broadband is atrocious. We now use Apple FaceTime and that is far superior.*

*[MK3, Male]*

Videoconference platforms enabled easier communication with loved ones at a time when it was convenient for both parties. For example, one participant felt Skype made conversing with loved ones much easier than using a landline:

*I think it’s far easier to use a Skype phone. I just use it because you are always going to be sitting there, waiting for the other person on the other end. And if you have just got a phone, you know ring them up, okay, if they’re not answering, they’re not answering, end of story sort of thing. Go back later.*

*[W7, Male]*

In addition to videoconference platforms, social media such as Facebook or communication platforms such as Viber enabled participants to communicate through text message and share photographs and videos:

*On my phone I use Viber, which is another… Actually, it runs off data; it doesn’t run off your ordinary phone. That’s very good; that’s instant messaging and photographs, pictures, all sorts.*

*[MK4, Male]*

These platforms enabled participants to share their social activities and remain connected. In some cases, participant chose not to share their own information, but enjoyed learning about others:

*“Facebook see what’s going on, for the gossip. You don’t have to participate; you can just be nosey”.*

*[W1, Female]*

Technology was valued among participants as a means of learning information that was relevant to them and as a way of communicating with family and friends. Perceiving it as useful for learning and sharing information served as a facilitator for engaging with technology among our participants.

#### 3.2.4. Feeling Secure

In addition to learning and sharing information, some participants felt that technology use offered a sense of security. In many cases, adult children who were concerned about the safety of their parents encouraged its use. Participants reported that adult children were concerned about driving long distances. For example, a participant from McBride stated the following:

*I got the cell phone because my kids kept thinking something was going to happen to me. I said, “Well you know if I have a breakdown on the highway, we managed for 70 years for God’s sake by just stopping someone and they’d help you. But now, “Oh my God they could murder you.” So, this was supposed to be a safety element to keep peace in the family.*

*[McB1, Female]*

While some participants adopted technology for safety when driving long distances, others did so in case of a health emergency:

*Oh, well, it was the bright idea of my son. I had a mini stroke. Oh, how old was I? 81, I think. I didn’t really know that I’d had one. My daughter was the one who took me into hospital and said, “You’ve got to be tested.” It did turn out that I did have a clot up behind my left ear somewhere, which affects this side of my face and my hand. Ever since, but they’re always frightened, my kids now, of a recurrence. So, my son gave me a cell phone, his old one, which I used right away, or more or less. About two years after I had the stroke, I think, they decided that I should have one, because I did get a few [brief] dizzy spells, but that was only the reason. So, now I just use it.*

*[McB2, Female]*

While originally motivated by health reasons and at the encouragement of her children, this participant adopted the technology for use in everyday life. In addition to mobile phone use for safety, some participants considered the usefulness of assistive devices. Wearable technology such as Lifesavers were considered as options if needed. A participant in Wales stated the following:

*“Yes, but there are a lot of people out there who actually use these Lifesavers.” *

*[W3, female]*

An additional participant in Wales spoke about the use of sensors to monitor movement:

*Or mats beside their bed, or mats in front, by the front door, in case they get out of bed and they shouldn’t. Or if they are by the front door and they shouldn’t be going out. So, there’s all that technology and not just the computer and the internet.*

*[W4, Female]*

While these types of technology were not currently being used by the participants, there was awareness that additional options for feeling secure could be available to them if needed.

When participants had easy access to technology, particularly the Internet, and felt that had some skill or understanding with regard to how to use technology, its use was feasible among our participants. They were largely interested in using technology to better understand their own health, and to maintain contact with friends and family. Further, feelings of safety and security served as a final facilitator of technology use.

### 3.3. Detractors of Technology Use

Participants also identified several detractors of technology use. They did not embrace all aspects of technology and identified several concerns. In particular, participants felt apprehensive about technology, lacked interest in technology, and found it difficult to learn how to use technology.

#### Feeling Apprehensive about Technology

Participants reported feeling apprehensive about using technology. A lack of understanding of how some technologies worked or how to use technology limited its use among our participants. For example, they expressed concern about being pressured to use technology that they did not feel comfortable with. One participant expressed fear at the rate that technology changed and her ability to keep up with it:

*I think the scary part now, isn’t it, everything is moving very fast in the IT world really? I do think that is an issue. Sometimes you just look in horror at the way that it’s moving. In a sense, you keep thinking, “I’ve got to keep up.” Some of it is way, way beyond anything that we would ever…yes, it’s just incredible, absolutely incredible. I find that quite exciting in some ways but staggering and frightening in other ways.*

*[MK4, female]*

While the general pace of development was overwhelming for participants, they also provided specific examples of instances where older adults were required to use technology they may not be comfortable with. Participants described being required to order prescriptions online rather than through the telephone, which they were accustomed to doing. One participant exclaimed,

People are being quite inextricably pushed towards using the internet. I mean I turned up at the doctors, only to pick up a prescription, which was unusual. And there was a notice up saying, “On December 1^st^…” And I had always ordered prescriptions… through the prescription line. That was changing from December 1^st^ …In actual fact you had to turn over because there was no longer a telephone prescription line.

*[W5, Female]*

Later in the conversation, the same participant expressed her concerns with forcing people to use technology they may not be comfortable with. With removal of the telephone prescription line, some older adults may be forced to go to their physician’s office in person, which may be difficult for them:

*In actual fact, what you are actually going to do is, you are going to handicap the less mobile, older people, who maybe might need a taxi or a bus and walk. Because there’s no bus right outside our surgery, and that’s not just our surgery, there are a lot. It is penalising people who are not able to use the internet.*

*[W6, Female]*

While using technology in new ways caused apprehension among participants, they also felt uncomfortable when pressured to upgrade their current technology. A participant in Regina felt pressure from her adult children to adopt more recent technology despite her satisfaction with her current capabilities:

*I have a cell phone, but I don’t text because the one I had that I bought six years ago has a, b, c, like. So, I am going to get a new one this year because after lots of pressure from my children, “Oh come on mum,” so I have a nice programme right that’s just phoning and it’s about $240 a year. I mean that’s 20 bucks a month without texting so that’s pretty good. I can still take pictures with it too.*

*[R2, Female]*

Participants also reported feeling apprehensive regarding asking for help for using technology because of barriers with regards to experience and language use:

*I went to some store and they made you feel like you’re stupid. They will not answer your questions and you don’t really know what to ask but you try to ask something, and they say….*

*[R1, Female]*

In addition, some participants were apprehensive about using social media platforms. Many participants wanted to maintain their privacy and were reluctant to use these platforms:

*I’ve got a Facebook account, but I don’t use it, because too much privacy and my wife has got thousands of friends who tell them that “Yes, I’ve had a cup of tea. I’ve eaten my sandwich.” The whole world lives like that.*

*[MK3 Male]*

Negative experiences with social media owing to lack of understanding of how it worked led to increased feelings of apprehension and, in turn, avoidance of these platforms:

*Facebook, I went onto for a short period, but, like a complete wally, I didn’t realise that unless you set up the privacy settings properly, everything you say is broadcast to the world. I fell out with my daughter quite badly over something […] “Right, I’m coming off that,” because there was so much garbage coming on.*

*[MK4, Male]*

Because of this apprehension, some participants took care to avoid sharing much detail about themselves when they did use social media:

*I do use Facebook because I’ve got a lot of friends who send me messages and say, “Like,” or, “Don’t like,” and small comments, but I never give any information about myself—I mean detailed information.*

*[MK2, Male]*

Similarly, a participant in McBride noted that while she enjoyed reading about others on Facebook, she avoided posting any information about herself because of concerns about privacy:

*Yes. I think about it and I am very careful about opening things because you can get a virus or whatever. I try to be very careful and yes, I am concerned about privacy. That is one reason I do not, very seldom will I answer on Facebook. I read what goes on, but I do not participate because of privacy.*

*[McB1, female]*

In addition to being apprehensive about social media use, participants reported apprehension about privacy and information sharing in terms of banking:

*Well you have to be very careful with your banking. I do my banking online which I find very, very convenient. But I am always kind of concerned about that, but I think, “Well there’s so little in there that who’d be interested anyway.” So, I feel I am pretty safe.*

*[McB1, female]*

Media reports of Internet scams also lent themselves to feelings of apprehension with regard to technology use. One participant talked about recent media reports and how they may affect older adults:

*I think it is interesting, and I think for older people when they hear so many different things, particularly about this week and some other banks—there were 900 scams in the last week in Santander, 900. That makes you panic, doesn’t it? I think for older people, I think IT is so important for them and yet they hear so many of the negatives, which are frightening for them. It’s that balance, isn’t it, of the two things of helping them to say, “Yes, if you do this, you’re okay, but there is this danger really”? *

*[MK4, female]*

A range of concerns were identified that led to feelings of apprehension among participants of this study, which impacted the degree to which they embraced technology in their day to day lives.

### 3.4. Lack of Interest in Technology Use

While in some cases, feelings of apprehension acted as deterrents of technology use, in other cases, there was a lack of interest in learning about or using technology. Again, social media arose as an example of a use of technology in which some participants lacked interest. A participant from Regina spoke about using email, but avoiding social media:

*I’m not involved in any of that social media, I find the email keeps me more than busy. I’m always getting people to want me to be on Facebook or whatever but there’s no way I have time for that.*

*[R1, Female]*

In some cases, in person, face-to-face communication was preferred over social media use, further highlighting a lack of interest in social media use:

*[...] I do not do Facebook. I decided a few years back I just didn’t want to spend any more time on the computer. I see social networks as being getting together with people like this and talking over a table more so than on Facebook. I find it too impersonal and that might just be my old-fashioned ways, but I would much rather talk to people face to face rather than on the computer.*

*[McB1, Male]*

Participants viewed technology use as anti-social, despite the opportunities it provided for keeping in touch with loved ones. As such, they had little interest in engaging with it.

One participant noted that, when riding the bus, she was surrounded by people using their devices:

*I’m sitting on the bus; every single person on that bus is doing this—every single person. They have forgotten how to communicate one with another. Communication verbally.*

*[MK6, female]*

This participant further lamented that a focus on devices minimized opportunities for face-to-face communication or conversation, and often its use was observed in inappropriate places:

*I think mobile phones are an [expletive], antisocial, because no matter where you go you’ve got people doing this, doing this. Even in front of me, a woman in front of me in Milton Keynes Theatre the other week had her mobile phone on, and I had to tap her on the shoulder and tell her to turn it off.*

*[MK6, Female]*

Similarly, participants did not always perceive wearable technology to be useful or of interest. A participant from Milton Keynes tried using a pedometer to keep track of physical activity, but soon lost interest: “I got fed up with it…According to what I was walking and doing, yes, you’re okay, but that was as much as it did.” [MK3, male]

Overall, some participants were not interested in technology until it was deemed to be of value. Social media, non-face-to-face communication, and fitness trackers did not provide tangible benefits versus the investments required to use them.

#### Difficulty Learning how to Use Technology

Learning how to use a new piece of technology or software can be difficult for anyone, and across our focus groups, our participants talked about the challenges they faced in learning how to use technology. For example, for some participants, switching between platforms was difficult and represented a significant learning curve.

*But I think the problem comes that there is nobody teaching people, and what to use, because I changed to Apple last year because my wife bought it for my birthday and that took me a learning curve. I think if somebody had never seen the technology, you’re lost. You get problems, like [when] my laptop went to Windows 10 that clashes with Norton’s, the utility software, so you have to take Norton’s off.*

*[MK3, Male]*

Furthermore, language or terminology posed a notable barrier to learning and understanding how to use technology. Clarifying the difficulty in terminology one participant commented,

*[…] it is really important to realise that older people in particular I think, and I hate to be ageist, but it is a different language that we didn’t learn in school. And anyone under 30 or 35 learned those terms and we don’t know.*

*[McB2, Female]*

Learning how to use peripheral devices such as a USB (Universal Serial Bus, a computer port which used to connect external equipment to a computer). An was also a concern. For example, one participant wanted to back-up photographs, using a USB stick, with additional memory, because,

My computer needs to be replaced and I’ve got to figure out a way to get all the pictures off of it and put them on disc so that I can get a new computer. So that’s my next thing that I have to do. I have a lot of pictures; I think they’re on my iPad now. I took them off my phone, they’re on my iPad and I have to connect the iPad to the computer and put more pictures on the computer before I get rid of them, before I put them on disc, so I have to do that. One thing I can’t do is put pictures; I haven’t learnt how to put pictures on a memory stick. That would be helpful I think […] I can put them on a memory stick, but I don’t know how to do that.

*[R3, Female]*

Moreover, one participant adopted a trial and error approach to learning to use technology, which enabled them to read an e-Book. Trial and error was preferred because asking children or grandchildren for assistance is not always easy. The terminology used by younger generations was unfamiliar to the participants. Further, the speed at which the instructions or demonstrations are delivered also caused the respective participant additional problems. The learning process unfolded like this,

I learned how to download books and I am really enjoying that as I go to bed at night and read, I thought, “I’m not going to like this to hold instead of a book.” But I find it is really, really great. And I am using that a lot. I am having some difficulty, I have to learn; well I am always learning, always learning but I have to do it trial and error because when I ask the grandchildren to show me something, they go so damn fast you can’t remember or follow what instructions they give you anyway.

*[MK1, female]*

One participant reported that she had to ask for assistance at an Apple store to gain access to her email account. The respective quote illustrates how non-technical users may become confused with terminology. Yet, asking for assistance from younger generations who (in some instances) may have grown up with technology and may find it difficult to relate on a personal level and communicate using terminology that would be understood and what can be accessed by different platforms:

I’ve got Apple... I took my iPad; I’ve got it with me. I took it all the way up to Apple because I wanted to get back into Google account. He couldn’t do it. I can’t understand why. Google is in America. Why do they not have somebody that talks to you? You have all these little bits of messages on the thing, like I was trying to do….I went to the Apple store and saw a man. In the end, he set me up an iCloud account with Google, but I want to get back onto emails; I want to get… I’ve got a new Google thing, but I can’t get in because it keeps saying, “Password is wrong, account is wrong,” and I’m, “Argh.” (Laughter).

*[MK5, female]*

While participants actively engaged with technology in a variety of ways, they also identified several factors that limited their use. Feeling uncertain about how to use technology, lacking interest in particular aspects of technology, as well as difficult learning how to use technology and understanding the language all detracted from further engagement with technology among our participants.

## 4. Discussion

This paper has presented findings from the Technology In Later Life (TILL) project—an international, multi-centered study, focusing on the use, behavior, and impact of technology by older adults residing in rural and metropolitan locations across two countries.

Quantitative data revealed a myriad of reasons (Figure 1) why adults aged ≥70 years adopt and engage technology into their lives, whether they are living in a rural or urban geographic location. Overall, over half of participants owned a mobile phone, though many more owned one in the UK (*n* = 18) than in Canada (*n* = 8), with just under ome-third owning an Apple iPad, while very few only used and owned a device to self-monitor or track one’s activity (e.g., Fitbit or pedometer). Canadian participants used social media more than UK participants. Those in rural locations of both UK and Canada use social media more than those in urban areas to stay connected with grandchildren or children.

Qualitative data analysis identified two primary themes: (1) facilitators of technology use, and (2) detractors of technology use; with seven subthemes, surrounding learning, access, sharing of information, positive and negative emotional wellbeing, justification of technology use, and competency of technology skills. While some participants spoke about their use and engagement with technology, initially through the workplace, for other respondents, this was not the case, and for some it was through intergenerational relationships with family members.

Participants perceive the Internet positively, especially those residing in a rural location; however, some participants did not see the point in social media platforms, and thus did not have a profile/account. This perspective was justified because there are more interesting things to do with their time, such as talking to people face-to-face, or the essence that social media can enable people to share information (i.e., photographs) without one’s permission.

While some participants chose to track or self-log, these participants enjoyed knowing how many steps they had walked per day, they did not always understand how to use the technology or how to access information on how to use the technology. For example, one participant was unsure what the ‘heart’ icon was used for on their mobile phone. Yet, the same participant noted their medication was logged. This suggests that the barrier to using mobile apps (mApp) is not necessarily a dislike for their use, but rather a lack of knowledge on ‘how’ to use the app. With improved access to information on the benefits of and instructions for use of an app, uptake by older adults may be increased. This is not to state that participants did not own devices; participants noted they owned many devices (see Table 6), with a mobile/cell phone been the most popular type and the least owned devices being a Fitbit/pedometer.

Despite identifying the facilitators of technology use, the factors that limited or discouraged them from using technology were also identified. In many cases, participants explained a simple lack of interest in technology use. In general, participants reported feeling apprehensive about the rate of technological change and some felt they had difficulty keeping up. Concerns about sharing too much information and the potential to be an invasion of privacy also lent itself to feelings of apprehension; especially if one did not realise that privacy settings were needed to ensure all information shared was kept between friends.

Privacy issues relating to older adults and their use of social media and technology have been understudied, yet across existing literature, we are starting to see how social media can play a positive element in life to maintain relationships with others [68,69]. However, this may not always be the case, as demonstrated by some participants in this study who chose not to use social media because it caused upset within families and friendship circles. Additionally, Table 4—Sharing Information illustrates the knowledge and experiences of study participants relating to self-logging, whether their friends or family members share data with them, and how often information is shared. Overall, many of the UK rural and urban participants reported to enjoy receiving this information.

Participants discussed how the use of technology may actually have a negative impact on society, resulting in an increase of social isolation. Furthermore, some participants perceived the use of technology was anti-social. In existing literature, researchers have been proposing the use of technology as a means of reducing isolation and enhancing social connectedness [25,70,71,72,73]. However, this was not the case for participants living in Milton Keynes—an urban area. In contrast, a participant from McBride, a rural location, discussed how she did not communicate with her family members as often as those who were using social media.

Enhancing social inclusion is integral to promoting positive ageing in place and reducing social isolation in later life. The findings from the TILL project show a positive trend towards age in place. For example, ensuring sound Internet services, provision, and infrastructure coupled with ICTs and associated technologies, such as social media platforms (i.e., Facebook), can facilitate individuals to share health-related information. Moreover, older adults have the opportunity to share photographs and narratives through different mediums (social media—Facebook), WhatsApp or mobile apps (mApps), which in turn may reduce social isolation. However, as evidenced here in the findings, our participants showed a mixed response to ICTs within their lives, owing to privacy concerns, interaction, learning new information, and jargon. To increase numbers of older adults who use technology in later life, in addition to making technology available, attention must be placed on developing age-appropriate education on strategies to use existing and new technologies. Participants in this study saw the benefits to using ICTs and digital devices; being able to share information; search for information; appeasing safety concerns of their children; and communicating with friends, children, and grandchildren. Therefore, ICTs can enable enhanced social inclusion and interaction, which in turn strengthens individuals, families, and communities [3].

Those participants in rural locations of both the UK and Canada use social media more than those in urban areas to stay connected with grandchildren or children. While participants explained, during the focus groups, concerns over privacy in social media; overall, all participants responded positively to hearing shared news and information from friends’ families, and nearly half of the participants had been using social media platforms such as Facebook for at least five years or more. Thus, this ability to stay digitally connected with friends, grandchildren, or children offers an additional means of communication. We can assume, based on previous research [10], those participants living in rural locations have less opportunity to regularly meet family members than those who live in metropolitan areas. This is particularly so for those family members who are geographically dispersed. Furthermore, while this work contributes to existing scholarly activity, it is also distinctive because of the study design and nature of the TILL study, and given the mere fact that social isolation and loneliness are key topics within the UK government; who has appointed Tracey Couch to the position of Minister for loneliness [74]. While it is beyond the scope of this paper, loneliness can and does play an integral role in ageing populations and could be exacerbated more so in rural locations than metropolitan. However, it should be noted that a person could still live in a metropolitan area and be lonely.

Primarily, participants in this study use technology in later life to communicate with family, in particular their adult children and grandchildren. Participants perceived technology as a digital ‘gathering place’, and technology is especially important when their family members are living in different geographic locations—be it overseas or in different parts of the country. Almost all participants in the urban sites used their computer for email, followed closely by using the internet to check facts, though this was much more common in urban areas of the UK and least common in rural areas of the UK. Social media/networking was mainly used in urban Canada and urban UK, with no rural participants in the UK and only 4 out of 10 in rural Canada using it. Given how technology was perceived by adults aged over 70 years, a range of purposes were identified for primary use, including reducing social isolation, safety reasons, sharing information, and undertaking tasks for community groups; one of the key differences between rural and urban participants was the rationale for owning a mobile/cell phone. Participants living in rural communities in both the UK and Canada used this type of technology for safety (when travelling to the city), while urban participants in both countries were more concerned about their privacy of data. Social media/networking is also one area where there is room for growth in connecting friends and family; the use of personal fitness trackers to enhance leisure, competitive virtual gaming (e.g., collecting digital badges or competing against friends in distances walked), and continued group sharing of information/communication [75] can all reduce social isolation.

While the TILL project follows similar and ongoing narratives in contemporary research, it is a distinctive initial study because of the study design—aims and objects, and focusing on a population of society that has garnered little attention from scholars. Yet, it is building on existing research [10,28,31,32,33,35,36,39,41,42,44,54,76]. On the basis of the findings of the work presented in this paper, we propose several recommendations aimed at policy makers, local and national organizations, stakeholders, and academics.

### 4.1. Recommendations

(1) Researchers, stakeholders, governments, and industry should focus on the strengths and opportunities, as opposed to focusing on simply overcoming the negative perceptions, attitudes, and barriers that ICT and associated technologies can bring to older adults, communities, and society. For example, the phenomenal development of mHealth Apps, which have enabled users to self-monitor their health and fitness [77]. While researchers and industry should demonstrate to individuals how such apps can enhance one’s life, rather than taking a deficit approach, how they plug a gap, would be appropriate given the narratives the older people gave in the focus groups;
(2) Training and education opportunities (age appropriate) should include peer-to-peer learning and support and support from peers, sharing information on mHealth Apps/mApps and Internet sites they use and why is often how using ICT is maintained among this age group. Having such support readily available in locations where computers can be shared, such as libraries or cafes, or where mobile phones can be looked at in comfort and safety are crucial;
(3) Create online support including both practical and emotional support by peers to include different terminologies, needs, and requirements;
(4) Employees across society who are working in computer shops or General Practitioner (GP) or primary care surgeries should consider how barriers or detractors to understanding technology specific language or technical jargon may impact the older adult’s abilities to engage with technology. Thus, integrating awareness training across varying environments should be considered as a government policy initiative, similar to existing awareness training conducted in employment. However, it is likely that a one-size-fits-all will not be suitable, and extensive consultation would be needed.

For further research to explore these findings in wider contexts, we suggest the following:

(1) Engage with different age cohorts to ascertain ICT and technology use, behaviour, and perception for future populations;
(2) Explore how intergenerational relationships work with older populations adopting and engaging with ICT across different regions/provinces and countries (Reference redacted for reviewing);
(3) Government agencies need to collect more detailed information on ICT use and ownership across all age cohorts, and not simply group all persons aged 65 years and older into the same age cohort. New questions may be added to existing surveys to reflect ICT and technological advances such as smart phones and mobile apps, and wearable health technologies to support age in place.
(4) Explore how ICT and associated technologies can facilitate positive age in place across the UK, Canada, and wider afield.

While we have proposed these recommendations, the TILL study does have limitations, yet we have identified areas for future work to grow and add new discussions to this work.

### 4.2. Limitations, Strengths, and Future Work

Several limitations should be considered regarding the TILL study. Each study site aimed to recruit 10 participants; however, the region of Regina was unable to recruit the full participant sample, and this may have been related to the recruitment processes allowed at that University. While this study took a geographic location approach, there is the limitation of not exploring ethnicity and gender, which may have also yielded further insights and perceptions. Future work should explore these factors regarding the digital divide, and technology adoption, building on the work of [17,18]. Although the sample size is small, the findings show a myriad of facilitators and detractors encountered and experienced by the respective participants.

The authors believe this is the first piece of work that has been conducted and culminates in this distinctive approach to data collection. Thus, the strengths and significance of this work not only contribute to furthering the discourse and debate surrounding technology use in later life in fields of gerontology, social sciences, and gerontechnology, but also has the capability of contributing to the field of public health. Given the national agenda and importance set by the UK government, loneliness can impact and carry associated health benefits to existing and future ageing populations.

Future work should include a lager sample size, with diverse populations including participants from indigenous populations, minority, and ethnic communities in addition to gender analysis. Encompassing additional locations from across the two countries would also provide a greater insight into the perception of ICTs in the lives of older adults, while also considering extending the sites to encompass countries from other geographic regions may provide different perspectives and insights. Furthermore, though we have offered several recommendations aimed at the non-academic community, we believe charities, stakeholders, policy makers, healthcare practitioners, and administrators should be aware of the issues facing ageing populations. Future work could explore the perceived perceptions encountered by patients and ICTs by employees, while also exploring the perception of ICTs within different settings (i.e., primary and secondary healthcare facilities) by staff. 

## 5. Conclusions

On the basis of the study design of the Technology In Later Life (TILL) study, the findings intersect at the fields of gerontology, geography, and gerontechnology. In particular, the authors have identified the barriers and challenges to using and adopting ICTs by adults aged 65+ years and over living in different geographic locations. The findings are distinctive based on the participants’ perceptions of ICTs and use of associated technologies, in a bid to provide older adults with different opportunities to ensure social connectedness and sharing of information, and to complement one’s hobbies and interests. The authors have proposed a series of recommendations aimed at widening this research to encompass wider stakeholders, policy makers, support networks (i.e., carers), and health practitioners; in a bid to ensure digital solutions are accessible for all in society and to facilitate positive age in place. Conducting this study in the UK and Canada has provided significant depth to understanding the milieu of the recruited participants in the Technology In Later Life (TILL) project, and offers future studies the opportunity to learn and build upon the findings and proposed recommendations presented here, which in turn offer a significant contribution to several scholarly fields.

## Figures and Tables

**Table 1:** Demographics of participants.

**a. Demographics of Participants by Country (UK and Canada)**			
**Demographics of Participants**	**Population (*n* = 37)**	**Canada (*n* = 16)**	**UK (*n* = 21)**
Mean Age ± SD	77.42 (6.41)	79.31 (5.86)	75.90 (6.56)
Age Range (*n*)	67.89	70–89	67–89
Gender			
Female	67.6 (25)	87.5 (14)	52.4 (11)
Male	32.4(12)	12.5 (2)	47.6 (10)
Employment Status			
Not currently employed (retired)	86.5 (32)	81.3 (13)	90.5 (19)
Currently employed	13.5 (5)	18.8 (3)	9.5 (2)
Marital status	54.3 (19)	50 (8)	57.9 (11)
Single/Widowed	34.3 (12)	25 (4)	42.1 (8)
Married/Living with partner	11.4 (4)	25 (4)	0
Other			
**b. Demographics of participants from rural and urban**			
**Demographics of Participants**	**Rural (*n* = 20)**	**Urban (*n* = 17)**	
Mean Age ± SD	77.50 (6.79)	77.31 (6.11)	
Age Range (n)	67–89	70–89	
Gender			
Female	70 (14)	64.7 (11)	
Male	30 (6)	35.3 (6)	
Employment Status			
Not currently employed (retired)	90 (18)	82.4 (14)	
Currently employed	10 (2)	17.6 (3)	
Marital status	40 (8)	73.3 (11)	
Single/Widowed	50 (10)	13.3 (2)	
Married/Living with partner	10 (2)	13.3 (2)	
Other			

**Table 2:** Type of computer used, location of use, and length of time used a computer by country (UK and Canada) and location (rural and urban).

Computer Use, Access, and Ownership	UK (*n* = 21)		Canada (*n* = 16)		Total
	Rural (*n* = 10)	Urban (*n* = 11)	Rural (*n* = 10)	Urban (*n* = 6)	
Computer Ownership					
Apple/Mac	1	1	2	1	5
PC	8	5	6	5	24
Unknown	1	2	3	-	6
Other	-	3	-	-	3
Physical Location of accessing Technology					
Own home	9	10	9	6	34
Friend’s house	7	1	-	-	8
Adult Child’s house	5	1	-	-	6
Public Building	5	3	-	-	8
Length of using a computer					
More than 20 years	3	4	7	3	17
Between 10–19 years	4	3	1	3	11
Between 5–9 years	3	2	1	-	6
Less than 1 year	-	1	-	-	1
Frequency of using a computer					
More than once a day	6	6	6	5	23
About once a day	-	2	1	1	4
More than once a month	-	1	1	-	2
Less than once a month	-	-	1	-	1
More than once a week	4	1	-	-	5

**Table 3:** Purpose for using a computer by country (UK and Canada) and location (rural and urban).

Purpose of Using a Computer	UK (*n* = 21)		Canada (*n* = 16)		Total
	Rural (*n* = 10)	Urban (*n* = 11)	Rural (*n* = 10)	Urban (*n* = 6)	
Email	6	10	7	6	29
Checking facts on the Internet	3	9	7	6	25
Drawing	-	1	-	1	2
Other	7	3	2	3	15
Word processing	7	8	5	4	24
Online shopping	6	8	6	2	22
Online banking	4	6	4	4	18
Social networking	2	3	4	4	13
Playing games	2	2	4	2	10
Database/Spreadsheets	0	5	2	1	8

**Table 4:** Social media use by country (UK and Canada) and location (rural and urban).

Use of Social Media	UK (*n* = 21)		Canada (*n* = 16)		Total
	Rural (*n* = 10)	Urban (*n* = 11)	Rural (*n* = 10)	Urban (*n* = 6)	
Do you use social media sites?					
Yes	0	5	4	4	13
No	10	6	6	2	24
Who introduced you to social media sites?					
Spouse/partner	-	-	1	-	1
Adult child	-	2	2	-	4
Friend	2	3	1	1	7
Relative	-	-	2	2	4
Other	-	-	-	1	1
Have you introduced anyone to social media?					
Yes	2	2	1	-	5
No	2	9	7	6	24
Years using social media sites:					
10 years	1	1	3	2	7
More than 5 years	3	3	1	1	8
More than 2 years	-	1	1	-	2
1 year or less	-	-	-	1	1
…<6 month or less	0	1	0	1	2
Frequency of using social media sites					
More than once a day	2	2	3	2	9
About once a day	1	1	1	1	4
More than once a month	-	1	1	-	2
Less than once a month	-	3	1	1	5
More than once a week	1	1	1	1	4
Social media to share information					
friends/family					
Yes	1	3	4	2	10
No	3	2	2	2	9

**Table 5:** Purpose for using social media (*n* = 37) by country (UK and Canada) and location (rural and urban).

Reasons for Using Social Media Platforms	UK (*n* = 21)		Canada (*n* = 16)		Total
	Rural (*n* = 10)	Urban (*n* = 11)	Rural (*n* = 10)	Urban (*n* = 11)	
Stay connected with friends	4	3	3	4	14
Stay connected Grandchildren/children	4	2	5	3	14
Share photographs with friends/family	2	4	4	2	12
Share information with friends/family	1	3	4	2	10
To keep up to date with news	4	2	1	1	8
To organize events	1	1	1	1	4
To take part in events	-	1	2	-	3
To express opinions	1	1	1	0	3

**Table 6:** Digital devices owned (*n* = 37) by country (UK and Canada) and location (rural and urban)

Digital Devices Owned	UK (*n* = 21)		Canada (*n* = 16)		Total
	Rural (*n* = 10)	Urban (*n* = 11)	Rural (*n* = 10)	Urban (*n* = 11)	
Mobile/cell phone	8	10	6	2	26
Apple iPad	2	3	1	4	10
Apple iPhone	2	0	3	3	8
Samsung phone	0	3	1	1	5
LG phone	0	1	2	4	5
Kindle/e-Book	3	1	1	0	5
Tablet	1	4	1	0	6
FitBit/Pedometer	2	1	-	-	3
Other	0	2	-	-	2

**Table 7:** Self-logging activities by country (UK and Canada) and location (rural and urban)

Sharing Information	UK (*n* = 21)		Canada (*n* = 16)		Total
	Rural (*n* = 10)	Urban (*n* = 11)	Rural (*n* = 10)	Urban (*n* = 11)	
**Common interests**	4	5	3	4	16
Because it’s fun	8	0	2	1	11
Inform people of my activities	1	1	2	4	8
To have other’s opinions	2	3	1	2	8
To make sure the recipient is thinking of me	7	1	-	-	8
To increase communication in friendships	2	2	0	3	7
To start/continue a conversation	4	1	0	2	7
I like to share information	1	1	2	0	4
To feel better	2	1	-	-	3
Build my confidence	1	0	1	0	2
Other	0	1	2	0	3

**Table 8:** Self-logging activities by country (UK and Canada) and location (rural and urban).

Self-Logging Activities	UK (*n* = 21)		Canada (*n* = 16)		Total
	Rural (*n* = 10)	Urban (*n* = 11)	Rural (*n* = 10)	Urban (*n* = 11)	
Friends or Family conduct self-logging activities?	6	3	3	0	12
Would you consider taking up self-logging?	5	2	0	1	8
Do you conduct self-logging on a Spreadsheet?	-	4	2	1	7
Do you conduct self-logging on a smart phone	3	2	0	1	6
Do you conduct self-logging on a PC?	-	5	-	-	5
Those self-loggers you know, do they share their data with you?	1	2	-	-	3
Do you conduct self-logging on a tablet?	1	1	-	-	2
Do you enjoy hearing this information	1	1	-	-	2
Do you conduct self-logging using traditional methods (pen/paper)?	-	-	1	0	1

**Table 9:** The facilitators and detractors of technology use.

Themes	
Primary Themes	Subthemes
***Facilitators*** of technology use	Technology learning opportunities Having access to technology Learning and sharing information Feeling secure
***Detractors*** to technology use	Feeling apprehensive about technology Lack of interest in technology use Difficulty learning how to use technology

## Supplementary Materials

The following are available online at https://www.mdpi.com/2227-9032/7/3/86/s1, Supplementary: A copy of the survey.
