# Supplementary material for: Older Adults’ Perceptions of ICT: Main Findings from the Technology In Later Life (TILL) Study
Source: Healthcare (Basel). 2019 Jul 4;7(3):86. doi: 10.3390/healthcare7030086 (PMC6787574; doi:10.3390/healthcare7030086)
Supplement: Supplementary file 1 [file healthcare-07-00086-s001.pdf]

Technology use by populations

We would like to know some basic details about your use of various technologies; social networking habits and sharing of information

Section A: Technology Use

Q1	Have you used a computer?	[ ] Yes<br>[ ] No
Q2	Do you own a computer	[ ] Yes<br>[ ] No
Q3a	Please state what type of computer do you own	[ ] Apple Mac<br>[ ] PC based<br>[ ] Other (please specify)<br>[ ] Don’t know
Q3b	Where do you use a computer? (please tick all that apply)	[ ] At own home<br>[ ] At a friend’s house<br>[ ] At a adult child’s house<br>[ ] At a public building (e.g. library, community centre)

***If you answered NO to Q3a, then please skip Q4-Q7, and turn to page 3**

Q4	How long have you used a computer? (please tick 1 answer)	[ ] More than 10 years<br>[ ] More than 5 years<br>[ ] More than 2 Years<br>[ ] For 1 year or less<br>[ ] 1 month or less
Q5	How frequently do you use a computer (please tick 1 answer)	[ ] More than once a day<br>[ ] About once a day<br>[ ] More than once a week<br>[ ] More than once a month<br>[ ] Less than once a month<br>[ ] I normally do not use a computer
Q6	How many hours a week do you use a computer? (please tick 1 answer)	[ ] 0-1 hours<br>[ ] 2-5 hours<br>[ ] 6-10 hours<br>[ ] 10+ hours<br>[ ] 11-20 hours<br>[ ] 20+ hours
Q7	What do you usually use a computer for (please tick all that apply)	[ ] Word processing<br>[ ] Drawing<br>[ ] Email<br>[ ] Playing games<br>[ ] Internet (checking facts)<br>[ ] Internet (Social networking, EG. Facebook)<br>[ ] Internet (banking)<br>[ ] Internet (purchasing)<br>[ ] Database/Spreadsheets<br>[ ] Recording data (health, finances)<br>[ ] Other (please specify)

Q8 Please briefly write how you learnt how to use a computer & its software ______________________

Q9 Have you played games?
[ ] Yes
[ ] No

*If you answered NO to Q9, then please skip Q10a-Q16, and turn to page 5

Q10 Do you own a video game console
[ ] Yes
[ ] No

Q11 Please state what type of game console(s) do you own? (if you own more than 1, please list all) ______________________

Q12 Do you have access to a computer that can be used to play games?
[ ] Yes
[ ] No

Q13 Do you own a handheld videogame console (e.g. Nintendo DS/ DS Lite/ Sony PSP
[ ] Yes
[ ] No

Q14 Please tick which kind of games you play
[ ] Adventure
[ ] Sport
[ ] Exergame
[ ] Strategy
[ ] Casual
[ ] Shooter
[ ] Role Playing
[ ] Action
[ ] Simulation
[ ] Puzzle
[ ] Other (please state)

Q15 Please write what your is your favourite game(s) to play ______________________

Q16 Please write how you learnt how to play a videogame ______________________

Section B: Internet use & Ownership

Q17	Do you have the Internet at home	[ ] Yes<br>[ ] No

***If you answered NO to Q17, please skip Q18-Q22, and turn to Q 23 page 5**

Q18	How much you pay per month for your Internet (which may include TV and phone line)	____________
Q10	How long have you used the Internet?	[ ] More than 10 years<br>[ ] More than 5 years<br>[ ] More than 2 Years<br>[ ] For 1 year or less<br>[ ] 1 month or less (I've just changed)
Q20	How frequently do you use the Internet?	[ ] More than once a day<br>[ ] About once a day<br>[ ] More than once a week<br>[ ] More than once a month<br>[ ] Less than once a month
Q21	Please write what you use the Internet for? (include all purposes)	[ ] Record video<br>[ ] Facebook<br>[ ] Google+<br>[ ] Take a photograph<br>[ ] Use Instagram<br>[ ] Pinterest<br>[ ] Snapchat, What's app, Other (please specify)<br>[ ] Twitter<br>[ ] Spotify, Deezer, Other (please specify)<br>[ ] Kindle<br>[ ] Foursquare<br>[ ] Netflix, BBC iPlayer, Other (Please specify)<br>[ ] Shazam<br>[ ] Games (please specifty)<br>[ ] Reading<br>[ ] Make a call, use Viber, use Skype<br>[ ] Send a text message<br>[ ] Access the Internet (Google)<br>[ ] Access Facebook<br>[ ] Access Twitter<br>[ ] Upload a photo to a social media site<br>[ ] Online Banking

[ ] Diary function
[ ] Weather report
[ ] Access news reports (BBC)
[ ] Use the alarm
[ ] Play games
[ ] Access GPS/Maps
[ ] Use as a note taker
[ ] Access/ send email
[ ] Access functional apps (e.g. Health, Trainline.com/ tube map – please specify)
[ ] Other (Please specify [ ] Email
[ ] Social media (e.g. Facebook)
[ ] Online shopping
[ ] Online banking
[ ] Searching for information
[ ] Booking holidays/flights
[ ] Booking events
[ ] Watching television
[ ] Streaming content
[ ] Downloading content
[ ] Other

Q22 Please write how you learnt to use the Internet including your behaviours (e.g. online banking) ____________________

Section C: Digital Device Ownership & Social Networking

Q23 Do you own any of the following digital devices (please tick all that apply)

[ ] Mobile phone
[ ] Blackberry
[ ] Apple iPad
[ ] Apple iPod
[ ] Apple iPhone
[ ] Kindle/e-book
[ ] Tablet
[ ] Fitbit
[ ] Other (please specify)

Q24 From the above list of devices & what you have selected, please write which you use frequently ____________________

Q25 What activities do you use your digital device for? (please tick all that apply)

[ ] Record video
[ ] Facebook
[ ] Google+
[ ] Take a photograph
[ ] Use Instagram
[ ] Pinterest
[ ] Snapchat, What's app, Other (please specify)

[ ] Twitter
[ ] Spotify, Deezer, Other (please specify)
[ ] Kindle
[ ] Foursquare
[ ] Netflix, BBC iPlayer, Other (Please specify)
[ ] Shazam
[ ] Games (please specifty)
[ ] Reading
[ ] Make a call, use Viber, use Skype
[ ] Send a text message
[ ] Access the Internet (Google)
[ ] Access Facebook
[ ] Access Twitter
[ ] Upload a photo to a social media site
[ ] Online Banking
[ ] Diary function
[ ] Weather report
[ ] Access news reports (BBC)
[ ] Use the alarm
[ ] Play games
[ ] Access GPS/Maps
[ ] Use as a note taker
[ ] Access/ send email
[ ] Access functional apps (e.g. Health, Trainline.com/ tube map – please specify)
[ ] Other (Please specify)

***If you answered NO to Q25, then please skip Q26-Q32, and turn to Q33 on page 7**

Q26	How long have you been using social networking sites? (please tick one answer)	[ ] More than 10 years<br>[ ] More than 5 years<br>[ ] More than 2 Years<br>[ ] For 1 year or less<br>[ ] 1 month or less (I've just changed)
Q27	How frequently do you use social networking sites? (please tick one answer)	[ ] More than once a day<br>[ ] About once a day<br>[ ] More than once a week<br>[ ] More than once a month<br>[ ] Less than once a month
Q28	Please tick why you use social media sites	[ ] To stay connected with friends<br>[ ] To stay connected with grand/children<br>[ ] To share information that I am interested in with friends/family<br>[ ] To share photographs with friends/family<br>[ ] To organize events<br>[ ] To partake in events/groups that I am interested in<br>[ ] To keep up to date with the latest news<br>[ ] To express my opinions and views<br>[ ] Other (please specify

Q29	Please tick who introduced you to social networking?	[ ] Spouse/partner<br>[ ] Adult child<br>[ ] Grandchild<br>[ ] Friend
Q30	Have you introduced anyone to social networking sites or apps?	[ ] Yes<br>[ ] No
Q31	Please tick who you introduced to social networking sites or apps to?	[ ] Spouse/partner<br>[ ] Adult child<br>[ ] Grandchild<br>[ ] Friend
Q32	Please write what type of social media site or App you introduce that person to?	____________________
Q33	Does anyone else access your digital devices?	[ ] Yes<br>[ ] No<br>[ ] Not sure
Q34	Please write which device(s) they have access to?	____________________
Q35	Please write who accesses your device(s)	[ ] Spouse/partner<br>[ ] Adult child<br>[ ] Grandchild<br>[ ] Friend
Q36	Please write what they use your device for?	____________________

Section D: Purchasing habits

Q37	Have you ever bought any of the following digital devices?	[ ] Mobile phone<br>[ ] Blackberry<br>[ ] Apple iPad<br>[ ] Apple iPod<br>[ ] Apple iPhone<br>[ ] Kindle/e-book<br>[ ] Tablet<br>[ ] Video game console<br>[ ] A handheld game console (e.g., Nintendo DS/Lite<br>[ ] Fitbit

[ ] Other

Q38	Please write what the reason was for buying these devices?	______________________
Q39	Where did you buy these devices from? (please tick all that apply)	[ ] Supermarket<br>[ ] A computer shop<br>[ ] A high street shop<br>[ ] A rental shop<br>[ ] Online (Amazon)<br>[ ] Online (specific store – e.g., Marks & Spencers)<br>[ ] Other (please specify)

Section E: Lifelogging/recording of data

Self-Logging – is a term where a person records a specific piece of information (e.g., mileage travelled in 1 day/holiday or a certain type of medication to be taken at a specific time or day)

Q40a We would like to know if you have previously or at present recorded this type of data. Please write what kind of items you have recorded in your daily life (past or present).

Life-logging is a term whereby, a person logs a variety of information on a daily basis relating to their physical activity. For example:

> Betty is 80 year old woman who enjoys the company of her children and grandchildren on a daily and weekly basis. She walks to her coffee mornings where she can catch up with her friends and to church on a Sunday morning. Recently, Betty found out one of her grandchildren – Jake (20 years old) who is an avid runner has started to track his own physical activity through the use of a Fitbit device.

Betty was fascinated with the Fitbit and wanted to know more information about this device. Jake showed his grandmother the simple interface which displays the time, number of steps taken, and how it can easily be attached to a piece of clothing. Betty asked Jake to buy her a Fitbit and to setup the synchronization on to her desktop computer, so she could update her progress on a daily basis.

Since wearing her Fitbit, Betty has now started walking everywhere (pending weather). She still attends her coffee mornings and weekly church services, but she also takes a walk after lunch and dinner, and she has started to share her progress with her friends, explaining to them, why she is has started doing this and how unobtrusive the Fitbit is in her activities of daily living.

We are interested in your knowledge and perception of life-logging. We are asking the following question to gauge the understanding, needs, and requirements, knowledge of older adults in relation to recording of data or logging specific activities.

Q40b	Do you undertake any self-logging in your life with a smart phone?	[ ] Yes<br>[ ] No<br>[ ] Not sure
Q40c	Do you undertake any self-logging activities with a tablet?	[ ] Yes<br>[ ] No<br>[ ] Not sure
Q40d	Do you undertake any self-logging activities with a PC?	[ ] Yes<br>[ ] No<br>[ ] Not sure
Q40e	Do you undertake any self-logging activities using a spreadsheet?	[ ] Yes<br>[ ] No<br>[ ] Not sure
Q40f	Do you undertake any self-logging activities using traditional methods (e.g. pen/paper)	[ ] Yes<br>[ ] No<br>[ ] Not sure

If you answered YES to Q40b-f, please go to Q41. If you answered NO to Q40b-f go to Q43

Q41	If you use a digital format for life-logging activities. Have you installed any of these 'apps' onto your digital device(s)? (please tick all that apply)	[ ] Runtastic<br>[ ] Strava<br>[ ] Fitbit<br>[ ] Foursquare<br>[ ] Pinterest<br>[ ] Health Apps (please specify all)<br>[ ] Work related Apps (please specify all)<br>[ ] Social Apps (please specify all)<br>[ ] Other (please specify all)

Q42	Have you heard of the following brands used for self-logging (please tick all that apply)	[ ] Fitbit<br>[ ] Jawbone<br>[ ] Garmin<br>[ ] Samsung<br>[ ] LG G<br>[ ] Garmin<br>[ ] No I haven't heard of any of the devices above<br>[ ] Other (please specify all)
Q43	Would you consider taking up Quantified Self (QS)-logging?	[ ] Yes<br>[ ] No<br>[ ] Not sure
Q44	If you answer YES to Q43 please select your reasons why	[ ] Because it's fun<br>[ ] To build my confidence<br>[ ] Because I like sharing information<br>[ ] To inform persons of my activities<br>[ ] To feel better<br>[ ] To have others' opinions<br>[ ] To make sure the recipient is thinking of me<br>[ ] To increase the amount of communication in friendship<br>[ ] Common interests<br>[ ] Start or continue conversations with friends<br>[ ] Other (please state)
Q45	If you answer NO to Q343please select your reason(s) why	[ ] It is not informative<br>[ ] It doesn't make sense to me<br>[ ] It has not real impact on my life<br>[ ] I don't know how to use this technology<br>[ ] I wouldn't know how to share this information<br>[ ] People might laugh at what I am doing<br>[ ] Because it is embarrassing<br>[ ] Persons might perceive me as being weird<br>[ ] This activity could take up too much of my time<br>[ ] I think it might be too expensive<br>[ ] I wouldn't know where to buy the equipment from<br>[ ] Other (please specify)
Q46	Do you know if any of your friends or family undertake self-logging activities	[ ] Yes<br>[ ] No<br>[ ] Not sure

If you answer YES to Q46 please go to Q47

If you answer NO to Q46 please go to Q51– Page 11

Q47	Please write who self-logs their data	[ ] Spouse/partner<br>[ ] Adult child

		[ ] Grandchild<br>[ ] Friend
Q48	For those who you know self-loggers, do they share their data/activity with you?	[ ] Yes<br>[ ] No<br>[ ] Not sure
Q49	Do they share their data with you on a regular basis?	[ ] Yes<br>[ ] No<br>[ ] Not sure

If you answer NO to Q48 & 49 please go to Q54, Page 12

Q50	How frequently do they share their data with you? (please tick 1 answer)	[ ] More than once a day<br>[ ] About once a day<br>[ ] More than once a week<br>[ ] More than once a month<br>[ ] Less than once a month
Q51	Do you enjoy hearing this information?	[ ] Yes<br>[ ] No<br>[ ] Sometimes<br>[ ] Not sure

If you answer YES to Q51 please go to Q52

If you answer NO to Q51 please go to Q53

Q52	If you answered **YES** to **Q51** Please tick why do you enjoy hearing this information?	[ ] Because it's fun<br>[ ] Because I find this information informative<br>[ ] Because they like sharing information<br>[ ] To inform me of their activities<br>[ ] It motivate me to undertake physical activity<br>[ ] I can provide my opinion(s) about their progress<br>[ ] I can show my support to the recipient during this physical activity<br>[ ] Our communication has increased and improved our friendship<br>[ ] Common interests<br>[ ] Start or continue conversations with friends<br>[ ] Other (please state)
Q53	If you answered **NO** to **Q51**<br><br>Please tick why you do not enjoy hearing this information?	[ ] It is not informative<br>[ ] It doesn't make sense to me<br>[ ] It has not real impact on my life<br>[ ] I don't undertake physical activity & it doesn't interest me<br>[ ] I might laugh at the person for not doing well<br>[ ] Because it is embarrassing<br>[ ] I have problems been motivated myself, I don't want to motivate others<br>[ ] I don't have the time to listen, read, watch or

speak about this information
[ ] I have my own problems
[ ] Other (please specify)

Section F: Sharing Information

Q54	Do you use digital devices to share information?<br>When using your digital device(s) or traditional methods, have you ever shared information (e.g. photo; exercise/ health data; personal feelings, websites?)	[ ] Yes<br>[ ] Sometimes<br>[ ] No
Q55	Do you use traditional methods for sharing information?	[ ] Yes<br>[ ] Sometimes<br>[ ] No
Q56	Either by digital or traditional methods, have you ever shared information (e.g. photo; exercise/ health data; personal feelings, websites?)	[ ] Yes<br>[ ] Sometimes<br>[ ] No

If you answer YES to Q54 please go to Q57

If you answer NO to Q54 please go to Q62 – page 14

Q57	Please write which digital device(s) or traditional approaches you have used to share information on?	________________
Q58	When did you start sharing your information? (please tick 1 answer)	[ ] More than 10 years<br>[ ] More than 5 years<br>[ ] More than 2 Years<br>[ ] For 1 year or less<br>[ ] 1 month or less
Q59	How frequently do you share your information? (please tick 1 answer)	[ ] More than once a day<br>[ ] About once a day<br>[ ] More than once a week<br>[ ] More than once a month<br>[ ] Less than once a month<br>[ ] I normally do not share information

Q60	Why are you sharing information?	[ ] Because it's fun<br>[ ] To build my confidence<br>[ ] Because I like sharing information<br>[ ] To inform persons of my activities<br>[ ] To feel better<br>[ ] To have others' opinions<br>[ ] To make sure the recipient is thinking of me<br>[ ] To increase amount of communication in friendship<br>[ ] Common interests<br>[ ] Start or continue conversations with friends<br>[ ] Other (please state)
Q61	What kind of information have you shared? (e.g., social networking sites; what's app, Viber or Twitter)	[ ] Photographs<br>[ ] Music (You tube; Spotify; Deezer)<br>[ ] Status updates (e.g. How I'm feeling today)<br>[ ] News articles<br>[ ] Job advertisements<br>[ ] Professional meetings (e.g. conferences)<br>[ ] Salutations (e.g. Happy Birthday to a friend or new job)<br>[ ] Personal health data (e.g. number of steps counted via a Fitbit)<br>[ ] Physical fitness (e.g. running route)<br>[ ] Other (please specify)
Q62	Would you consider sharing information? (e.g. on Social media sites, specific health/app related websites)	[ ] Yes<br>[ ] No<br>[ ] Not sure
Q63	What concerns do you have about sharing your information (please tick all that apply)	[ ] Information being stolen<br>[ ] Persons wouldn't be interested<br>[ ] I don't know how to share this information<br>[ ] It is my data and I don't want to share it<br>[ ] Persons might laugh at me<br>[ ] Because it is embarrassing<br>[ ] Sharing could make others evaluate me negatively<br>[ ] Sharing could result in a loss of control over who knows this about me<br>[ ] Sharing could result in an increase in telephone or mail solicitations from 3rd party companies<br>[ ] Other (please specify)

Section G: Demographic Information

Q64	What is your age?	
Q65	What is the annual household income	
Q66	Do you have private health insurance?	[ ] Yes<br>[ ] No

If you have answered YES to Q66 please go to 67

If you have answered NO to Q66 please go to Q68

Q67	Would you consider wearing a Fitbit device if your insurance company agreed to provide you with a year discount for accessing your information?	[ ] Yes<br>[ ] No<br>[ ] Not sure<br>[ ] Maybe, but I would like to know more information
Q68	What is your gender?	[ ] Male<br>[ ] Female
Q69	What is your marital status?	[ ] Single<br>[ ] Married/Living with partner<br>[ ] Widowed<br>[ ] Other
Q70	Are you currently employed?	[ ] Yes<br>[ ] No<br>[ ] Retired (please state your previous occupation)
Q71	What level of education have you gained? (highest gained, please only tick 1 answer)	[ ] GCSE/O-level/ High School equivalent<br>[ ] A-Level/ College equivalent<br>[ ] Higher Education – BSc/BA (Hons)<br>[ ] Higher Education – MSc/MA<br>[ ] Higher Education - MPhil<br>[ ] Higher Education – PhD<br>[ ] Teaching Certification – PGCE<br>[ ] Other (please state)
Q72	Who do you live with?	[ ] Live alone<br>[ ] With spouse or partner<br>[ ] With adult child<br>[ ] With grandchild<br>[ ] With family, friend or acquaintance<br>[ ] Other (please state)
Q73	What are you living arrangements?	[ ] At home<br>[ ] Long-term care<br>[ ] Assisted living

Q74 What is the type of community do you live in?

[ ] Rural (<2500)
[ ] Small town (2501-10000)
[ ] City suburb (10001-50000)
[ ] Metropolitan city (50001>)

Q75 How do you rate your current physical activity (please select 1 answer)

**Never**	**Infrequent**	**Neutral**	**Once a day**	**More than once a day**
1	2	3	4	5

Q76 How do you rate your current health status (please select 1 answer)

**Very unhealthy**	**Somewhat unhealthy**	**Neutral**	**Somewhat healthy**	**Very healthy**
1	2	3	4	5

Thank you for completing the survey.
